# Research on a machine learning-based adaptive and efficient screening model for psychological symptoms of community correctional prisoners

**DOI:** 10.1038/s41598-024-60181-9

**Published:** 2024-04-30

**Authors:** Zhifei Xu, Zhigeng Pan, Yan Wang, Yichao Zhang, Pengfei Leng

**Affiliations:** 1 School of Public Health, Hangzhou Normal University, Hangzhou, 311121 Zhejiang China; 2 Department of Information Technology and Management, Zhejiang Police Vocational Academy, Hangzhou, China; 3 School of Artifical Intelligence (School of Future Technology), Nanjing University of Information Science & Technology, Nanjing, China; 4 Institute of VR and Intelligent System, Hangzhou Normal University, Hangzhou, China; 5 School of Information Engineering, Hangzhou Medical College, Hangzhou, China

**Keywords:** Public health, Psychological symptoms, Community correctional prisoners, Machine learning, Adaptive testing, Screening model, Public health, Psychology, Machine learning

## Abstract

Community correction institutions in China frequently employ the Symptom Checklist-90 (SCL-90) and the health survey brief (SF-12) as primary tools for psychological assessment of community correctional prisoners. However, in practical application, the SCL-90 Checklist faces issues such as complex item numbers, overall low cultural level of the subjects, and insufficient professional level of the administrators. The SF-12 health survey brief, as a preliminary screening tool, although only has 12 questions, to some extent simplifies the evaluation process and improves work efficiency, it is prone to missed screening. The research team collected 17-dimensional basic characteristic data and corresponding SCL-90 and SF-12 data from 25,480 samples of community correctional prisoners in Zhejiang Province, China. This study explored the application of multi-label multi-classification algorithms and oversampling techniques in building machine learning models to delve into the correlation between the psychological health risks of community correctional prisoners and their characteristic data. Inspired by computerized adaptive testing (CAT), we constructed an adaptive and efficient screening model for community correctional prisoners through experimental comparisons, based on the binary relevance algorithm with sample oversampling. This screening model personalize the assessment process by dynamically matching participants with the most relevant subset (s) of the nine dimensions of the SCL-90 based on their individual characteristics. Thus, adaptive dynamic simplification and personalized recommendation of the SCL-90 scale between question groups were achieved for the specific group of community correctional prisoners. As a screening tool for psychological symptoms of community correctional prisoners, this model significantly simplifies the number of questions compared to SCL-90, with a simplification rate of up to 65%. However, it achieves this simplification while maintaining excellent performance. The accuracy reached 0.66, with a sensitivity of 0.754, and an F1 score of 0.649. This innovation simplified the assessment process, reduced the assessment time, improved work efficiency, and enhanced the ability to judge the specificity of community correctional prisoners population. Compared to the SF-12, although the simplification rate and accuracy of the model are slightly lower than those of the SF-12, the sensitivity increased by 42.26%, and the F1 score improved by 15.28%. This means the model greatly reduces the possibility of missed screening, effectively preventing prisoners with abnormal psychological or mental states from losing control due to missed screening, and even committing suicide, self injury, or injuring others.

## Introduction

### The necessity of psychological assessment of prisoners

There are obvious abnormalities in the psychological health status of the special group of prisoners. Xu Yanchun et al. investigated the psychological health status of prisoners by using the SCL-90 scale. The results indicated that the scores of 9 symptom factors among prisoners were significantly higher than the national norm. Among them, the four most prominent factors were psychosis, somatization, depression, and anxiety^1^. The survey conducted by Liu Suzhen et al. found that the overall average score and positive factors of SCL-90 among community prisoners were higher than the norm of the Chinese general population^2^. The average scores of community inmates, especially in factors such as compulsion, somatization, depression, paranoia, and hostility, were significantly higher than the norm of the Chinese general population^2^. According to the classification method in the Chinese National Psychological Counselor Tutorial, a survey conducted by the Guangdong Prison Administration revealed that 41.36% of prisoners exhibited significant psychological problems, of which 9.01% presented serious problems, and 7.08% were diagnosed with psychological or mental diseases by medical diagnosis or judicial appraisal^3^.

The occurrence of criminal behavior is closely associated with adverse psychological symptoms among offenders^4^. The occurrence of criminal behavior is the external manifestation of the criminal’s psychological activities. The frustration-aggression theory proposed by psychologist Rosenzweig offers insights into the psychological mechanisms underlying violent recidivism^5^. Erikson regards self-identity crisis as a significant cause of juvenile delinquency or other behavioral problems^6^. Scholars suggest that criminal behavior is closely related to the psychological personality of criminals. The transformation of psychological personality is closely linked to the purpose, motivation, means, and attitude of committing a crime^6^.

If the psychological problems of prisoners cannot be accurately evaluated, and there are cases of false screening (false positive) or missed screening (false negative), it will result in a lack of timely and scientific management and correction for the group. On the one hand, it will seriously interfere with normal regulatory reform work, and even lead to accidents that endanger regulatory safety. For example, Wang Huanqin et al. found that psychological health status and sleep quality were risk factors for suicide among prisoners^7^. On the other hand, because of their own psychological, personality, and social factors, a small number of released prisoners may commit crimes again^5^. For example, some violent criminals are of higher risk due to their antisocial personality, paranoid personality disorder, delusions of persecution and other psychological problems. The integration of criminal psychology and personality structure shows a steady tendency to commit crimes. If their criminal psychology and personality cannot be fundamentally corrected, they are susceptible to violent recidivism when they are stimulated by family or social conflicts after released from prison^5^. According to statistics from the Ministry of Justice, China’s recidivism rate is currently at the world's average level. However, in major criminal cases involving malicious violence, up to 70% of cases are caused by the recidivism of released prisoners^8^.

Therefore, it is an important part to accurately evaluate the mental activities and personality characteristics of criminals by psychological measurement for the psychological correction work of prison and the community correctional center^4^. All theories and methods of psychological measurement have been applied in the assessment of risk behavior among inmates. The conclusion of psychological assessment not only helps to grasp the psychological structural characteristics and changes of prisoners, but also helps to identify tendencies such as suicide, violence, and escape. Additionally, it can predict the likelihood of reoffending^9^. In the United States, the results of psychological test have been effectively applied in judicial practice and become an important basis for the criminals’return-society schemes such as vacation, commutation, parole and so on^10^. It is of great significance in reducing supervision risks, adjusting correction plans, and improving effects by means of carrying out psychological health risk assessment regularly and learning about the true emotional status of the prisoners which contributes to reduce the rate of recidivism, and protect the safety of people's lives and property^11^.

### The primary tools for conducting psychological assessments of prisoners

Psychological assessment scales are commonly used tools for conducting psychological health screenings, widely employed due to their extensive applicability, straightforward administration, adherence to scientific standards, and informative results^12^. These scales serve as important instruments for examining the psychological structure and states of prisoners, evaluating the effectiveness of rehabilitation and predict their behavior tendency in the future. Currently, the Symptom Checklist-90 (SCL-90), Eysenck personality questionnaire, 16 Personality Factor Questionnaire (16PF), and Chinese Offender Psychological Assessment-Personality Inventory (COPA-PI) are the primary tools utilized for psychological assessments of offenders in China^4,13^. Among them, the Symptom Checklist-90 (SCL-90) stands out for its strong reliability and validity. It is widely used by domestic and international scholars in educational and psychological research. It has become one of the prevalent and effective tools employed by the Chinese prisons and community correctional institutions for psychological evaluations, with test results reflecting individual personalities and psychological deficiencies^14^.

### The main issues in current scale assessment

Although the combination of interviews with psychological scale assessments is a traditional method in the field of mental health, it has limitations and challenges when dealing with the special population of prisoners and their prison environment. These shortcomings are mainly manifested in the following aspects:

1. The first issue is the large and complex number of scale (questionnaire) items, which makes it difficult to ensure the authenticity and objectivity of the assessment results. Researchers always aim to obtain more comprehensive information from participants by designing more questions^15^. For example, the Symptom Checklist-90 (SCL-90) includes nine categories of psychological measurement indicators, such as somatization, depression, and anxiety, corresponding to nine factors, totaling 90 items. However, the special group of inmates not only have poor compliance, but also have a lower average cultural level. Surveys indicate that over 60% of the incarcerated population have an education level below junior high school^16^. The scale’s large number of questions makes it difficult for prisoners to accurately understand the meaning of the questionnaire items, and they often lack the patience to complete numerous items. Excessive evaluation time causes subjects to develop a sense of boredom and passive coping, often resulting in psychological measurement work, becoming formalistic, and reliability and validity being difficult to guarantee^13,17^. The current version of the SCL-90 questionnaire used in China was translated by Wang Zhengyu^18^, and consists of 90 items that take approximately 20 min to complete. In the prison environment, where inmates typically have lower levels of education and compliance, completing the questionnaire may be a difficult task^17^.
2. The second issue concerns the time-consuming and laborious statistical analysis of assessments, which results in delayed feedback. The large number of questions not only makes the organization and management of assessments extremely inconvenient but also makes statistical analysis of assessment data more time-consuming and laborious, making it difficult to ensure the timeliness of feedback.
3. The third issue concerns the widespread use of transplant evaluation tools, which are often not tailored to the specific situation of different prison units, resulting in a lack of targeted evaluation. The most commonly used psychological assessment tool in China is the translation of foreign tools, which may have cultural differences. Furthermore, mental health issues vary significantly across regions, demographics, educational backgrounds, and age groups. This variability somewhat limits the effectiveness of assessment tools^19^.
4. The lack of highly skilled assessors in prisons and community correctional institutions is a significant issue, as it makes it difficult to ensure the accuracy and objectivity of psychological assessment results. Psychological measurement is a highly specialized activity of identification and judgment, and the accuracy of measurement conclusions largely depends on the abilities, professional qualities, and extensive practical experience of the examiners. This requires examiners to have a background in multiple disciplines such as psychology, physiology, statistics, etc., and to undergo education and training, as well as passing a unified psychological measurement examination to obtain a professional qualification certificate before being competent for this job after a period of practice. Therefore, in practice, high-level examiners are still scarce. Most of the police officers engaged in psychological measurement only hold certificates as psychological counselors, or have only undergone short-term training in psychological measurement, with many being "self-taught." Furthermore, some prisons and community correctional facilities employing police officers for psychological measurement experience rapid turnover, lacking stability or longevity. This situation results in a generally low overall professional quality of police officers engaged in psychological measurement in prisons and community correctional facilities, making it difficult to ensure high accuracy of psychological measurement conclusions^17^.

### Development trends in psychological scale (questionnaire) assessments

Currently, researchers are developing simplified and efficient psychological assessment tools to address the challenges in psychological scale (questionnaire) assessments. With the rapid advancement of information science technologies such as data mining, big data analysis, and artificial intelligence, researchers are attempting to deeply integrate these advanced technologies with traditional paradigms of psychological research. The aim is to revolutionize the research logic, methodology, and performance of traditional psychological measurement tools, thereby overcoming the limitations of existing psychological assessment methods.

Machine learning algorithms are increasingly used in the fields of psychological disorders and clinical psychiatry, and have shown significant effectiveness in simplifying lengthy and time-consuming psychological assessment scales (questionnaires). The use of machine learning algorithms to simplify psychological scales can shorten evaluation time and identify more correlations within the scale, laying the foundation for the promotion and application of the scale in various scenarios^20^.

In research related to simplifying psychological assessment scales using machine learning algorithms, researchers are primarily concerned with the assessment scale for autism spectrum disorder (ASD) and the classification of autism. By using support vector machine (SVM) and other algorithms, the simplification rate of such scales can exceed 55%, and the classification accuracy can exceed 90%^21–24^.

In China, Ma Yantao and other researchers utilized a machine learning algorithm to simplify the evaluation of affective disorders (ADE) from 118 items to 18 items. And the accuracy of the simplified scale for the differential prediction of patients with major depression, bipolar disorder and healthy individuals reached 93%, 96% and 99.6%, respectively^25^ in 2019. In 2020, Liu Jinming applied a machine learning algorithm to simplify the Symptom Checklist-90 (SCL-90) under the physical examination scene. By using the method of predicting the score between and within the test groups, seven of the 10 test groups of the SCL-90 scale could be deleted. The prediction accuracy between the test groups could reach 75.9% -81.3%, and each test group could be reduced from 6-13 to 3-7^26^. In 2021, Sun Qike simplified the Minnesota multiple personality test using a machine learning algorithm, while maintaining a specificity and sensitivity of 85% for the test results of the simplified scale^27^. In 2022, Shang Mingyue developed a data-driven method for optimizing the SCL-90 psychological evaluation scale using machine learning algorithms. The study also investigated the impact of the size of the psychological evaluation data on the simplification of the scale. The method addressed the issue of reduced accuracy in prediction models when dealing with small amounts of data and established a minimum threshold for the application of the scale simplification method^28^.

Currently, simplified version of SCL-90, such as the brief symptom inventory (BSI)^29^, SCL-27^30^, have been proved to be effective in screening^31,32^. But the simplification process of such scales is to directly develop a simple version based on the relevant theories of psychology, or using factor analysis and other methods to generate a fixed short version scale. It is also difficult to ensure the comprehensiveness and specificity of the screening, as there is no in-depth exploration of the relationship between the scale evaluation and the subject group and individual trait data based on the differences in mental health issues among groups and individuals. In practical application, another important issue about the scale is the timeliness^33^. Most studies on the norm of the scale are data from 10 years ago and are not applicable to the needs of mental health assessment in the current specific scenario^26^.

In recent years, computerized adaptive testing (CAT) has emerged as a new trend in the field of psychological measurement. CAT is a test method based on computer algorithm. It is the product of the full combination of psychological measurement, computer technology and modern educational technology^34^. CAT differs from the traditional test of "one thousand people one volume". Its purpose is to provide an optimal test for each candidate^35^. CAT allows the computer to act as the chief examiner, and calculate the trait level of the subjects in real time. Based on the trait level of the subjects, it selects personalized test questions from the question bank to ensure the most suitable topic is chosen under the current topic selection strategy. This technology can rapidly and precisely measure the target characteristics of subjects, particularly in large-scale tests such as censuses and selections. It saves financial, human, and material resources, reduces the burden on subjects, and protects the question bank, thereby extending the test's service life.

### Main contributions of this study

In this work, we present our main contributions:

1. Firstly, we drew inspiration from the traditional Chinese medicine concept of 'treating before illness' and the practices of computerized adaptive testing (CAT). Secondly, we have fully utilized big data and artificial intelligence technologies. We have developed an adaptive, efficient, and cost-effective screening and evaluation model to assess the psychological health risks of community correctional prisoners. The goal is to improve the predictive, preventive, and alert capabilities of community correctional institutions' security management. This model aims to enhance the pertinence of correctional and rehabilitation work for prisoners and improve the effectiveness of correctional and rehabilitation work. Finally, it will help prevent and reduce the recurrence of crimes by released prisoners, especially in reducing the occurrence of major and malignant criminal cases, and maximize the protection of people's lives and property safety.
2. The proposed model offers convenient, efficient, and cost-effective advantages that help conserve resources such as manpower, materials, and finances in psychological assessment work within community correctional institutions. It reduces the burden on subjects, facilitates high-frequency, large-scale psychological health risk surveys conducted by community correctional institution management personnel, and enables more accurate screening of psychological symptoms.
3. The proposed model enhances the group specificity of screening and evaluation, and deeply explores the correlation between the psychological health risks and demographic characteristics of community correctional prisoners, including gender, age, education level, management level, types of crimes, recidivism status, criminal history, and other group trait data. This approach provides more personalized and effective solutions for rehabilitation.

## Materials and methods

### Data collection

#### Data collection

The research group collected 17-dimensional basic trait data (Supplementary Information 1. and Supplementary Information 2.) of 25480 samples of community correction prisoners in Zhejiang Province, China, and the corresponding Symptom Checklist-90 (SCL-90) and Health Survey Short Form (SF-12) data. These data were collected through the standardized community correction digital management platform of the Zhejiang Provincial Department of Justice, covering the period from January 2020 to December 2020. The 17-dimensional characteristics mainly include age, sex, treatment level (general control, strict control), whether adult, education level, dmicile (urban or rural), whether there are infectious diseases, whether belongs to the following three categories (unemployed individuals, those without relatives to rely on, individuals without a place to live), whether there is a criminal record, crime type, supervision time, whether there is recidivism, whether there is anti-government tendency, whether there are five kinds of involvement (terrorism, cults, drugs, gangs, and gun trafficking), whether there are four histories (drug use history, escape history, suicide history, police assault history), correction status (in correction, released from the status of correction), occupation before arrest. The SCL-90 traditional scale obtained 9 kinds of psychological measurement indicators: somatization, obsessive-compulsive symptoms, interpersonal sensitivity, depression, anxiety, hostility, terror, paranoia, and psychosis. Due to the incomplete basic information registered in some judicial offices, the samples with missing values in the basic information were removed and matched, resulting in a total of 25,214 sample data.

Due to the privacy and compliance issue of patients, it is difficult to collect a large number of medical data, especially the data of specific groups. The research group has invested a lot of manpower, material and financial resources in the construction of this data set (Supplementary Information 3.).

#### Data declaration and ethical review

The research design has been approved by the Ethics Research Committee of the Zhejiang Community Correction Management Bureau. This study was carried out in accordance with the Declaration of Helsinki, and all procedures were carried out in accordance with relevant guidelines and regulations. The Committee waived the requirement of informed consent for this study because the researchers only access the database for analysis purposes, and all personnel, including patient data, are desensitized, and there is no conflict of interest among personnel of each unit.

### Data preprocessing

The pretreatment of tabulated data described in the paper includes missing value imputation, outlier detection and removal and data standardization, as follows:

#### Missing value imputation

Missing values refer to situations where the values of certain features or variables in a table are missing or not recorded. In machine learning modeling, handling missing values is crucial^36^. Choosing appropriate filling methods can improve the predictive performance of the model, making the data more complete and reliable^37^. In this study, there were some missing values in the raw data we used, and most of the missing values were filled in by manually tracing the raw materials. For a small amount of other missing values such as age and other quantitative data, we use mean interpolation to fill in, as the mean can represent the central trend of the data and help maintain its distribution.For qualitative data such as crime types, we use the median to fill in, which is a better choice because it can reduce the impact of extreme values while maintaining the order and level of the data^38^.

#### Outlier detection and removal

Outliers refer to data points that are significantly different from other data points or deviate from the normal range. Outliers may have adverse effects on data analysis and modeling, so they need to be eliminated or handled. To ensure the accuracy and reliability of the data, we carried out outlier detection and elimination. We use the Rajda criterion to deal with outliers. The process takes the given confidence probability of 99.7% as the standard, and is based on the standard deviation of 3 times of the data column. The abnormal data row greater than the value is deleted, and when the residual error vb of the measured value xb is greater than 3 times σ, outliers should be eliminated.

$$\left| {vb} \right| = \left| {xb - x} \right| > 3\sigma .$$

#### Data standardization

Data standardization is to transform the data of different scales and ranges into a unified standard scale to eliminate the influence of dimensions and make different features comparable. In the stage of data preprocessing, we normalize the numerical features from minimum to maximum. By linearly mapping the values of each feature to the range of 0 to 1, we eliminate the differences of different feature scales and make them comparable.

### Simplification of multi-label classification algorithm

Based on symptom checklist-90(SCL-90), this study constructed an adaptive scale (between question groups) simplification screening evaluation model based on multi-label classification algorithm, and used Health Survey Short Form(SF-12), a primary screening tool commonly used by community correction management institutions, as a simplified baseline method for comparative analysis.

We used the multi-label classification model for scale (between question groups) simplification to analyze the risk degree of individuals in nine categories of psychological measurement indicators, and simplified the scale structure based on the risk distribution. The goal of scale simplification is to simplify the questions, make the scale more readable and easy to understand, and help readers get core information and insight more quickly. During the process of scale simplification, it is necessary to make trade-offs and decisions according to the data and the needs of the audience to ensure that enough information is retained while maintaining simplicity and clarity.

**Figure 1:**
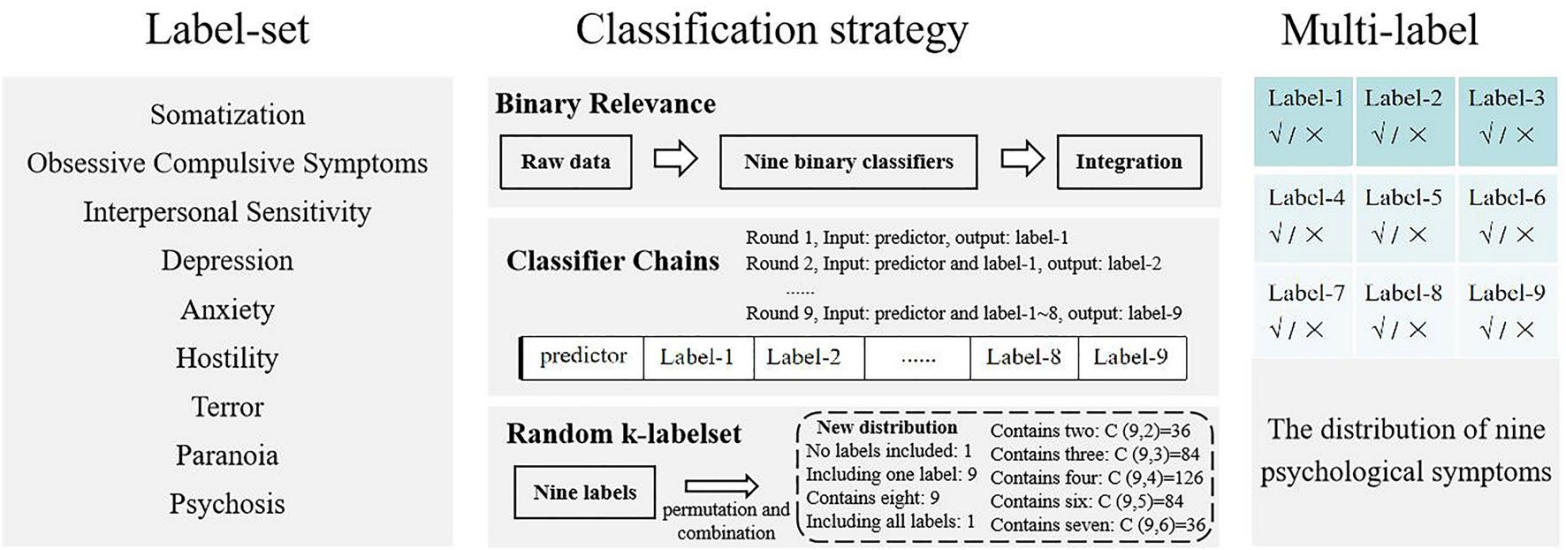
Multi label classification algorithm.

The basic principle of the multi-label classification algorithm (as shown in Fig. 1 and Table 1) is to recognize the association between features and labels by learning historical data, so as to predict new labels. It can integrate the results of multiple tags, find the association between multiple tags, and solve the multiple conflicts that may exist in the multi-tag classification problem, so as to effectively improve the accuracy of classification. It can also help us quickly identify features, thus reducing the time of classification.

1. *Binary relevance* (first-order, y tags are independent of each other). It is a problem transformation method. The core idea is to decompose the multi-label classification problem. BR is simple and easy to understand. When there is no dependent relationship between Y values, the effect of the model is good.
2. *Classifier* *chains* (high-order, y tags are interdependent). Its principle is similar to the BR conversion method. In this case, the first classifier is trained only on the input data, and then each classifier is trained on all previous classifiers in the input space and chain. A certain number of binary classifiers can be combined into a single multi-label model to explore the correlation between multiple targets.
3. *Rakle (*random k-labelsets, high-order, y tags are interdependent). It can divide the original large tag set into a certain number of small tag sets, then use RF to train the corresponding classifier, and finally integrate the prediction results. RakeID is a high-order strategy algorithm, which can mine the correlation of multiple tags according to the size of the tag subset.

**Table 1:** Multi-label multi-classification algorithm.

Method	Strategy	Algorithm
Transformation of problems	First order strategy	Binary relevance
	Second order strategy	Calibrated label ranking
	High order strategy	Rakle (random k-labelsets)

Problem conversion: fit data to algorithm.

For the latter two algorithms, if there is a clear dependency between tags, the generalization ability of the final model is better than that of the model constructed by binary relevance. The problem is that it is difficult to find a more suitable tag dependency.

### Model training

The core principle of oversampling method is to increase some samples in the category with fewer samples to achieve category balance. SMOTE is the representative algorithm of the oversampling method. In the process of modeling, SMOTE (Synthetic Minority Over-sampling Technique) is used to solve the problem of category imbalance. SMOTE increases the number of minority samples by synthesizing new minority samples, to balance the unbalanced data set.

Because the total number of samples collected is sufficient, the training data adopts 5-fold cross-validation to prevent the model from overfitting and increase the robustness of the model. The extracted feature data is randomly divided into five parts, four of which are used for training, and one part is retained as test data. The above process is repeated five times, using different test data each time. Then the results of these five times are summarized, and the average value is taken as the estimation of the algorithm performance index. Five cross-validation is a popular algorithm choice at present.

### Model evaluation

#### Comparison

In this paper, SF-12 was used as a comparison tool. SF-12 is a commonly used health questionnaire survey tool, which is used to assess the health status and quality of life of individuals. SF-12 is a simplified version derived from the SF-36 questionnaire, which retains the core concepts and dimensions of SF-36. However, it reduces the number of questions and improves the efficiency of questionnaire implementation. The simplicity and efficiency of the SF-12 questionnaire make it a common tool in large-scale epidemiological research and clinical practice. It can be used to evaluate the health status of different groups and the effect of health intervention, and compare the health differences between different groups.

#### Overall evaluation index

If all SCL-90 subscales of the actual sample are diagnosed as risk-free, the sample is defined as a negative sample. If any subscale test is risky, the sample is defined as a positive sample. Similarly, if all the sub-tags predicted by the multi-label model are 0, the sample is negative. If there is any positive sub-tag, the sample is positive:

If the actual 9 labels are all negative, the mental state is healthy and marked as a negative sample.

If one of the actual 9 labels is positive, the mental state is unhealthy and marked as a positive sample.

Similarly, if all of the predicted 9 tags are negative, the mental state is healthy and the tag is negative.

If one of the predicted 9 tags is positive, the mental state is unhealthy and marked as a positive sample.

According to the actual mental state and the predicted value, the confusion matrix (as shown in Table 2) is drawn, which is composed of the following four important definitions: true positive (TP), false positive (FP), false negative (FN) and true negative (TN).

**Table 2:** Confusion matrix.

	Forecast 0	Forecast 1
Actual 0	TN	FP
Actual 1	FN	TP

The overall effect of the model is evaluated by the following indicators, including accuracy, sensitivity, specificity and F1. The relevant measurement standards are as follows:

$${\text{Accuracy }} = \left( {{\text{TP }} + {\text{ TN}}} \right)/\left( {{\text{TP }} + {\text{ TN }} + {\text{ FP }} + {\text{ FN}}} \right),$$

$${\text{Sensitivity }} = {\text{ TP}}/\left( {{\text{TP }} + {\text{ FN}}} \right),$$

$${\text{Precision }} = {\text{ TP}}/\left( {{\text{TP }} + {\text{ FP}}} \right),$$

$${\text{F1}} = {2} \times {\text{Sensitivity}} \times {\text{Precision}}/\left( {{\text{Precision}} + {\text{Sensitivity}}} \right).$$

#### Multi label classification evaluation index

In the multi label classification problem, accuracy_Score, Hamming loss and 0-1 loss related evaluation indicators can be based on the prediction results of a single tag or the overall prediction results.

Accuracy_Score is the correctly predicted score (default) or count. In multi-label classification, the function returns the subset precision. If the whole set of predicted tags of the sample matches the real tag combination, the subset accuracy is 1. Otherwise, it is 0.

Hamming loss: Hamming loss measures the prediction accuracy of the model for each label, that is, the ratio of the number of labels with average prediction errors to the total number of labels. It calculates the prediction result of each tag and returns a value between 0 and 1. The smaller the value, the more accurate the prediction is.

0-1 loss is a common classification loss function, which is used to measure the prediction error of the classification model. It takes 1 when the prediction is wrong and 0 when the prediction is correct, so it is named 0-1 loss.

#### Simplification rate assessment

Simplification rate refers to the proportion of the simplified scale to the original scale, which can be used to evaluate the degree of simplification of the scale. Scale simplification refers to simplifying the structure of the original scale by reducing the number of items, deleting redundant or unnecessary items, or merging multiple items. The simplification rate of the scale can be calculated in the following way: simplification rate (number of simplified items/original number of items) ×100%. In other words, the simplification rate based on the multi-label model is calculated as follows: simplification rate (the number of sub-labels predicted to be negative)/(the total number of samples).

### Institutional review board statement

The Ethics Committee of the Zhejiang Community Correction Management Bureau has waived the informed consent requirement for this study, as researchers accessing the database is only for analytical purposes, including patient data, which is desensitized, and there are no conflicts of interest between personnel in each unit. The research design has been approved by the Ethics Research Committee of the Zhejiang Community Correction Management Bureau. This study was conducted in accordance with the Helsinki Declaration, and all procedures were conducted in accordance with relevant guidelines and regulations.

## Experimental results

### General description

The study participants included 25480 community correctional prisoners. After missing value imputation, outlier detection and removal, and data standardization pretreatment, 9297 valid samples were obtained (as shown in Table 3), and 1860 samples (a quarter of the total samples) were selected as the validation set.

**Table 3:** General characteristics of the study population.

Characteristics	n (%) or mean (SD)
Age, years	43.48 (12.24)
Sex	
Male	7113 (76.51%)
Female	2184 (23.49%)
Treatment level	
General control	8131 (87.46%)
Strict control	1166 (12.54%)
Education level	
Illiteracy	240 (2.58%)
Elementary school	1427 (15.35%)
Middle school	3546 (38.14%)
Senior school	1693 (18.21%)
Vocational schools	310 (3.33%)
Junior college	1385 (14.90%)
University	639 (6.87%)
Other	57 (0.62%)
Domicile	
Urban	2578 (27.73%)
Rural	6719 (72.27%)
Is there any criminal record	
Yes	1106 (11.90%)
No	8191 (88.10%)
Type of crime	
Disrupting social management order	3531 (37.98%)
Disrupting the rank of market economy	2124 (22.85%)
Property violation	1651 (7.42%)
Infringement of civil rights	690 (17.76%)
Corruption and bribery	140 (1.51%)
Endanger public security	970 (10.43%)
Other	191 (2.05%)
Supervision time	
Probation	144 (1.55%)
Execution outside of prison	2 (0.02%)
Within 1 year	1092 (11.75%)
1 to 3 years	6220 (66.90%)
3 to 5 years	1752 (18.84%)
More than 5 years	87 (0.94%)
Whether there is recidivism	
Yes	111 (1.19%)
No	9186 (98.81%)
Whether there are five kinds of involvement (terrorism, cults, drugs, gangs, and gun trafficking)	
Yes	189 (2.03%),
No	9108 (97.97%)
Whether there are four histories (drug use history, escape history, suicide history, police assault history)	
Yes	184 (1.98%)
No	9113 (98.02%)
Correction status	
In correction	7393 (79.52%)
Released from the status of correction	1904 (20.48%)
Occupation before arrest	
Private entrepreneurs and individual workers	911 (9.80%)
Clerk	2199 (23.65%)
People’s organization	827 (8.90%)
Unemployed personnel	1788 (19.23%)
Retired personnel	134 (1.44%)
Other	3438 (36.98%)

### Experimental results

The experimental results, as shown in Table 4, indicate that the first-order Binary Relevance model, after sample oversampling, achieved the best overall performance and sensitivity F1, is superior to the other two high-order methods of transformation strategy-based multi-label classification algorithms. Compared to the SCL-90 questionnaire, this model simplified 65% of the items while maintaining good performance, with an accuracy of 0.66, sensitivity of 0.75, and F1 score of 0.65. Therefore, a machine learning model based on this algorithm can be constructed to explore the association between the psychological health risks of community correctional offenders and the 17-dimensional target trait data. Using the idea of CAT for reference, according to their trait level, we can personalize and recommend the most suitable one or more sub-scales for the subjects from the nine sub-scales of symptom checklist SCL-90. Thus, the self-adaptive dynamic simplification and personalized recommendation of the SCL-90 scale (between question groups) for the community correctional prisoners are realized.

**Table 4:** Comparison of performance indicators of different multi-label algorithm models and baseline control scales.

Performance index	Classifier chain	RakelD	Binary relevance	SF-12
Tp	279	176	584	341
Fn	496	599	191	303
Fp	61	37	442	223
Tn	1024	1048	643	993
Accuracy	0.701	0.658	0.660	0.71
Sensitivity	0.360	0.227	0.754	0.530
Precision	0.821	0.826	0.569	0.600
F1	0.501	0.356	0.649	0.563
Accuracy_score	0.585	0.584	0.543	–
Hamming loss	0.208	0.199	0.182	–
0–1 loss	0.415	0.412	0.457	–
Simplification rate	0.817	0.894	0.651	0.867

In recent years, grassroots community correctional institutions have often adopted the Short Form-12 (SF-12) health survey questionnaire as a screening tool for efficient and convenient mental health risk assessment. This study includes SF-12 as a control analysis, simplifying the original 90-item SCL90 questionnaire to 12 items, with a simplification rate of 86.7%. The accuracy was 0.71, sensitivity was 0.53, and F1 score was 0.56 (as shown in Table 4).

Comparatively, although the adaptive and efficient screening model obtained in this study had a slightly lower simplification rate and accuracy than SF-12, its sensitivity and F1 score was significantly superior to SF-12.

**Figure 2:**
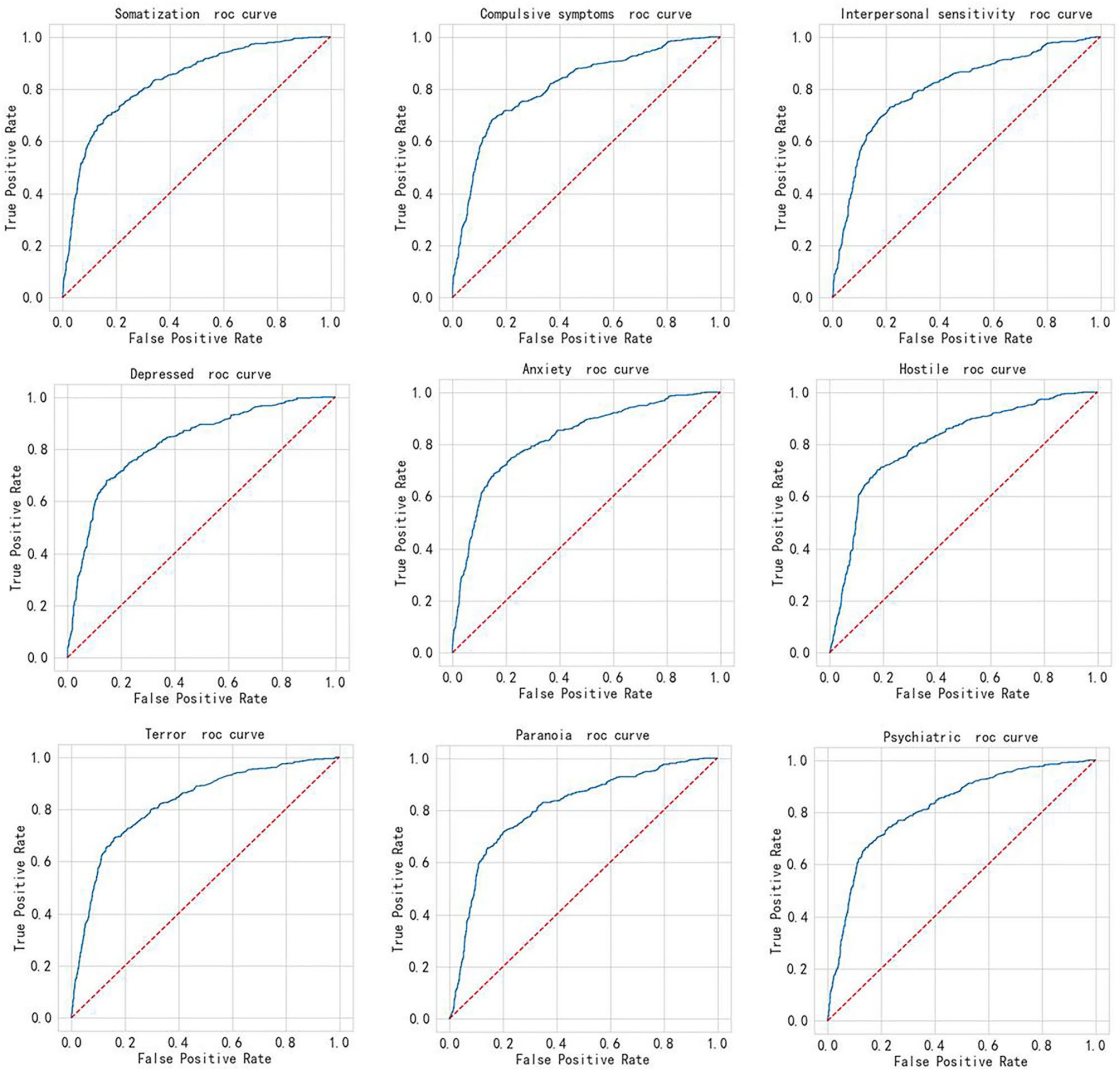
ROC comparison of evaluation model for different labels.

Furthermore, we designed a stratified experiment to explore the model's assessment capabilities for the 9 symptom factors. As shown in Table 5 and Fig. 2, the experimental results revealed that the model demonstrated good sensitivity for three high-risk psychological symptoms among community correctional offenders, including compulsion, anxiety, and depression.

**Table 5:** Comparison of performance indicators of evaluation model for different labels.

Label	Accuracy	Auc	Sensitivity	Specificity
Somatization	0.71	0.832	0.82	0.67
Obsessive compulsive symptoms	0.456	0.725	0.94	0.2
Interpersonal sensitivity	0.374	0.733	0.94	0.21
Depression	0.623	0.793	0.84	0.56
Anxiety	0.652	0.787	0.8	0.61
Hostility	0.609	0.735	0.74	0.59
Terror	0.722	0.775	0.72	0.72
Paranoia	0.578	0.701	0.72	0.55
Psychosis	0.688	0.777	0.75	0.68

## Discussion

### Overview

The study innovatively applied multi-label multi-classification algorithms and oversampling techniques to construct machine learning models for assessing the psychological health risks of community correctional prisoners. We have achieved adaptive dynamic simplification between scales (between question groups) and personalized recommendation of sub-scales (question groups) for the SCL-90 scale in the specific scenario of assessing the psychological health risks of community correctional prisoners. This not only simplifies the number of questions, reduces testing time, and improves work efficiency, but also enhances group and scenario specificity. The performance of the model was validated and evaluated by using SF-12 as a simplified baseline method as a control.

For the study of psychological symptoms, it is difficult to collect a large number of medical data due to the privacy and compliance issues of patients, especially the special group of community correction prisoners. Through a survey conducted over the past 2 years, this study collected 25214 samples' 17 dimensional basic trait data and corresponding SCL-90 and SF-12 assessment scales data using the Digital Management Platform of Community Correction of Zhejiang Provincial Department of Justice. Due to the low cultural level and poor compliance of this special group, 9297 valid samples were obtained after missing value filling, outlier elimination,and standardized data preprocessing, of which 1860 samples (a quarter of the total sample) were selected as the validation set.

In recent years, in order to carry out psychological health risk assessment on a large scale efficiently and conveniently, grass-roots community correction institutions usually use the SF-12 scale (Short Form-12) as a primary screening tool. This study included it as a control analysis.In comparison, the simplified rate and accuracy of the adaptive and efficient screening model obtained in this study are slightly lower than those of SF-12, but the sensitivity and F1 values are significantly better than those of SF-12. Community correction institutions pay the most attention to safety management and try to reduce the rate of missing screening in the psychological health risk assessment of prisoners, which can prevent prisoners with abnormal psychological and mental states from losing control due to missing screening, or even committing suicide or harming others. Therefore, the key indicator of sensitivity is of great importance for the mental health risk assessment of prisoners in community correction. Obviously, the group adaptability of the adaptive and Efficient screening assessment model is better than that of SF-12, and it is more suitable for the large-scale preliminary screening of the psychological health of community correction prisoners.

### Social significance

Firstly, it helps to address the issue of inaccurate psychological condition assessment caused by factors such as community correctional offenders' negative coping or resistance tendencies. It is more convenient for them to accurately understand the meaning of scale questions, improve their patience and confidence in completing scale questions. Thus, enhancing the authenticity and objectivity of evaluation results.

Secondly, it helps to crack the problem of insufficient timely detection of psychological abnormalities. This helps to achieve the prediction, prevention, and early warning of psychological abnormalities in community correctional prisoners. Traditional psychological correction methods usually intervene when prisoners exhibit abnormal behaviors such as violating rules and regulations. Efficient and rapid screening and adaptive personalized recommendations can detect psychological abnormalities and disciplinary signs of prisoners more timely and accurately. This also helps to scientifically distinguish the categories of mental health risks. Early intervention and correction can achieve the effect of "solving problems in the bud".

Thirdly, it facilitates the community correctional institutions in conducting screening and assessment work conveniently, efficiently, and cost-effectively. It helps overcome challenges such as insufficient number of assessors and professional expertise, saving resources in terms of finances, personnel, and materials invested in psychological assessment work.

Fourthly, it helps to exert the effect of correcting and shaping the heart. Due to the diversity and particularity of the negative psychology and personality of prisoners, traditional "person recognition" experiences and educational correction methods cannot meet the requirements of improving their personality. Therefore, scientific and accurate personalized assessment, classification warning, and targeted correction of psychological conditions of prisoners are particularly important for exerting the "correction and shaping" effect.

In summary, the screening model aims to make the psychological health risk assessment of community correctional prisoners more efficient and convenient, which helps to predict, prevent, and warn the psychological health risks of prisoners, thereby ensuring regulatory safety. The second is to enhance the pertinence of correction and rehabilitation work for community correctional prisoners, which helps to prevent and reduce recidivism, especially in reducing the occurrence of major and malignant criminal cases, and maximizing the protection of people's lives and property safety.

### Problems and deficiencies

This study has the following limitations:

1. The issue of missed screening has not been fully resolved yet. Despite achieving a sensitivity of 0.754 in the screening model, which represents a significant improvement compared to the sensitivity of 0.530 in the current SF-12 screening tool used by correctional management agencies, missing screening still exists. Among the 1860 items in the validation set, 633 items were predicted incorrectly, of which 442 items without disease were predicted to be sick (false screening (False Positive)), and the remaining 191 items with disease were predicted to be disease-free (missed screening (False Negative)). Due to the need for safety management, the community correctional institutions attaches great importance to the problem of missed screening. In this study, 191 missing samples in the validation set were statistically analyzed, including 62 cases of anxiety (32.46%), 57 cases of depression (29.84%), 145 cases of compulsion (75.91%), 87 cases of interpersonal sensitivity (45.55%), and 38 cases of coexistence of anxiety and depression (19.9%). It can be seen that the proportion of obsessive–compulsive symptoms is the highest in the missed samples, followed by interpersonal sensitivity. For community correction management institutions, due to the positive correlation between anxiety, depression, and the risk of suicide and self harm^39^, which pose serious threats to supervision, high-risk samples with coexisting anxiety and depression in the missed screening samples need to be highly valued.
2. The sample data is not complete enough. The data used for the study are from the sample data of Zhejiang community correction institutions in 2020, which can not accurately represent the whole situation, nor can it carry out more valuable time series analysis.
3. Simplification within the table (within the question group) is yet to be studied. Based on the dynamic simplification between tables (between question groups) achieved in this study, we can also explore the relationship between questions more deeply, and further assign weights to questions representing different characteristics, so as to obtain a simplified scale with higher accuracy and efficiency.

## Conclusion

This study is based on the 17-dimensional trait data of 9287 community correctional prisoners in Zhejiang Province and the corresponding data of the SCL-90 scale. By comparing and appling three types of multi-label and multi-classification algorithms combined with oversampling methods, the association between the psychological health risk of this special group and the target trait data is excavated and analyzed. Based on the above research, we have constructed an adaptive and efficient screening model for the psychological risk of community correctional prisoners.

Compared with the SCL-90 scale, this model simplifies 65% of the questions while maintaining good performance. Compared with the Health Survey Short Form-12 (SF-12), which is usually used by grassroots community correctional institutions as the primary screening tool, although the model’s simplification rate and the accuracy rate is slightly lower, the two most important indexes valued by community correctional institutions, the sensitivity and F1 score, are significantly better than the SF-12. This model is more suitable for the special group.

The intelligent assessment of psychological health issues is an interdisciplinary problem at the intersection of artificial intelligence, psychology, and medicine, highlighting the importance of in-depth interdisciplinary communication and collaboration^40^. In the future, we will continue to make full use of the industry advantages of the judicial administration teaching and research units where the researchers are located, and collect larger-scale and more time-series sample data with the help of a uniformly deployed management information system. In order to get more accurate model, we will use different algorithms to further analyze and combine the revised norm data, so as to further promote the intelligence and Individualization of psychological test for the prisoners.

### Supplementary Information

Supplementary Information 1.

Supplementary Information 2.

Supplementary Information 3.

## Data Availability

The data supporting the results of this study was provided by the Zhejiang Provincial Community Correction Administration, but the availability of these data is limited. These data were used under the permission of the current study and are therefore not publicly available. However, with the permission of the Zhejiang Provincial Community Correction Management Bureau, data can be obtained from the corresponding author/[Zhifei Xu, Zhigeng Pan]. We have provided some sample data that have been desensitized in the supplementary documents for the convenience of the researchers’ review.

## References

[1] Chunyan Xu, Hongzhong Q (2007). Investigation and analysis of mental health of prisoners. Chin. J. Health Psychol..

[2] Liu S, Zhu J, Fan Q (2006). A survey on the psychological health status of community prisoners. Psychol. Sci..

[3] Bureau GPA (2009). Investigation report on the psychological status of prisoners in Guangdong Province. Res. Crime Correct..

[4] Yuan Z, Ming Y (2022). Application and research trends of criminal psychological measurement in China. J. Shandong Police Acad..

[5] Zhou J (2023). Research on the Prevention of Violent Recidivism: Taking 50 Cases of Violent Recidivism as an Example.

[6] Erikson EH (1998). Identity: Youth and Crisi. Translated by Sun Mingzhi.

[7] Huanqin W, Chai Hongyan Xu, Guangjun WY, John Z (2017). The relationship between suicide risk, psychological health, and sleep quality among prisoners. Chin. J. Health Psychol..

[8] Linlin J (2012). Cause analysis and countermeasures of recidivism of released prisoners. J. Henan Police Coll..

[9] Dahua L (2000). New Compilation of Criminal Psychology.

[10] Pan H (1994). History of Foreign Prisons.

[11] Shuang L, Suqiong Q, Bin Z (2019). Psychological stress status and treatment countermeasures of male prisoners. Res. Crime Correct..

[12] Preti A, Carta MG, Petretto DR (2019). Factor structure models of the SCL-90-R: Replicability across community samples of adolescents. Psychiatry Res..

[13] Duojin S (2003). New progress in criminal personality psychometric research. Chin. J. Forensic Sci..

[14] Fu D (2008). Psychological characteristics of prisoners and psychological crisis intervention strategies. J. Hunan Public Secur. Coll.

[15] Mai, Y., Wang, M., Liu, T. Simplification of the “Reactivity-Initiative Aggression” scale based on item response model and factor analysis. In *Summary of the 21st Chinese Psychological Society Conference* (2018).

[16] Huiwen L (2012). Difficulties and countermeasures of employment skills training of prison prisoners. Legal Syst. Soc..

[17] Mingshu Xu (2021). Analysis of the current situation of prison abuse psychological measurement. Res. Crime Correct..

[18] Zhengyu W (1984). Symptom checklist (SCL-90). Shanghai Arch. Psychiatry.

[19] Fu X, Zhang K, Chen X, Chen Z (2021). Report on National Mental Health Development (2019–2020).

[20] Caiyi L, Yuan Y, Wenjun H (2014). Development, Reliability and Validity of Mental Health State Image Projection Test. China Journal of Health Journal of Health Psychology.

[21] Kosmicki JA, Sochat V, Duda M (2015). Searching for a minimal set of behaviors for autism detection through feature selection-based machine learning. Transl. Psychiatry.

[22] Wall DP, Dally R, Luyster R (2012). Use of artificial intelligence to shorten the behavioral diagnosis of autism. PLoS One.

[23] Duda M, Haber N, Daniels J (2017). Crowd sourced validation of a machine-learning classification system for autism and ADHD. Psychiatry.

[24] Washington P, Paskov KM, Kalantarian H (2020). Feature selection and dimension reduction of social autism data. Pac. Symp. Biocomput..

[25] Ma Y, JiJ HY (2019). Implementing machine learning in bipolar diagnosis in China. Transl. Psychiatry.

[26] Jinming L (2020). Simplification and Application of Symptom Checklist Based on Machine Learning.

[27] Qike S, Wentian D, Ke W (2021). Research on the validity of simplified MMPI scale based on machine learning. J. Qingdao Univ. (Nat. Sci. Ed.).

[28] Shang, M. Research and application of data-driven optimization method for psychological evaluation scale. University of Jinan (2022).

[29] DerogatisL R, Nick M (1983). The brief symptom inventory: An introductory report. Psychol. Med..

[30] Hardt J, Egle UT, Kappis B (2004). Symptom checklist SCL-27. Psychother. Psychosom. Med. Psychol..

[31] Alvir JMJ, Schooler NR, BorensteinM T (1988). The reliability of a shortened version of the SCL-90. Psychopharmacol. Bull..

[32] Prinz U, NutzingerD O, Schulz H (2013). Comparative psychometric analyses of the SCL-90-R and its short versions in patients with affective disorders. BMC Psychiatry.

[33] Tong, H. Twenty years’ vicissitude in China-Mainland: SCL-90 and its norm. In *The Sixth Annual Academic Conference of the International Chinese Association for Applied Psychology* (2009).

[34] Cheng X (2011). New item selection criteria of computerized adaptive testing with exposure-control factor. Acta Psychol. Sin..

[35] Meijer RR, NeringM L (1999). Computerized adaptive testing: Overview and introduction. Appl. Psychol. Meas..

[36] Johnson WH, Xu W (2015). A preliminary study on cleaning up erroneous data and filling in missing values in a medical record. IFAC-PapersOnLine.

[37] Rios R, Miller RJH, Manral N (2022). Handling missing values in machine learning to predict patient-specific risk of adverse cardiac events: Insights from REFINE SPECT registry. Comput. Biol. Med..

[38] Weed L, Lok R, Chawra D (2022). The impact of missing data and imputation methods on the analysis of 24-hour activity patterns. Clocks Sleep.

[39] Yanfang T, Caiyan Y (2021). The relationship between alexithymia and suicide risk in prisoners: The mediating role of depression and anxiety. Chin. J. Health Psychol..

[40] Liming J, Xuetao T, Ping R, Fang L (2022). A new type of mental health assessment using artificial intelligence technique. Adv. Psychol. Sci..

