# Supplementary material for: Research on a machine learning-based adaptive and efficient screening model for psychological symptoms of community correctional prisoners
Source: Sci Rep. 2024 Apr 30;14:9890. doi: 10.1038/s41598-024-60181-9 (PMC11061180; doi:10.1038/s41598-024-60181-9)
Supplement: Supplementary file 1 — Supplementary Information 1. [file 41598_2024_60181_MOESM1_ESM.pdf]

Data annotation and explanation

1.Age:Numerization
2.Sex:Male:1,Female：2
3.Whether adult:Yes:1,No:2
4.Management level:Ordinary:1,Strictly：2
5.Education level:Illiteracy:1,Primary school:2,Middle School:3,High School:4,Technical secondary school and technical secondary school:5, Junior college:6, Undergraduate course:7
6.Domicile:Urban:1,Rural area:2
7.Is there any criminal record:Yes:1,No:2
8.Is there any infectious disease:Yes:1,No:2
9.Whether there are three categories of people (homeless, unemployed, no relatives to rely on):Yes:1,No:2
10.Supervision time:Probation:1,Execution outside of prison:2,Medical parole:3,Within one year:4,One to three years:5,Three to five years:6,More than five years:7
11.Type of crime:Disrupting social management order:1, Disrupting the rank of market economy:2, Property violation:3,Infringement of civil rights:4,Corruption and bribery:5,Endanger public security:6,other:7
12.Whether it is a recidivist or not:Yes:1,No:2
13.Correction status:Correcting:1,Relieve correction:2
14.Occupation before arrest:Private entrepreneurs and individual workers:1,Clerk:2,People's organization:3,Unemployed personnel:4,Retired personnel:5, Other:6
15.Anti government tendencies:Yes:1,No:2
16.Is it related to terrorism, cults, drugs, gangs, and gun trafficking:Yes:1,No:2
17.Is there a history of drug use, escape, suicide, and assault on police officers:Yes:1,No:2
