# Supplementary material for: Research on a machine learning-based adaptive and efficient screening model for psychological symptoms of community correctional prisoners
Source: Sci Rep. 2024 Apr 30;14:9890. doi: 10.1038/s41598-024-60181-9 (PMC11061180; doi:10.1038/s41598-024-60181-9)
Supplement: Supplementary file 3 — Supplementary Information 3. [file 41598_2024_60181_MOESM3_ESM.pdf]

The Statement of Data Availability

The data supporting the results of this study was provided by the Zhejiang Provincial Community Correction Administration, but the availability of these data is limited. These data were used under the permission of the current study and are therefore not publicly available. However, with the permission of the Zhejiang Provincial Community Correction Management Bureau, data can be obtained from the corresponding author/ [Zhifei Xu,Zhigeng Pan]. We have provided some sample data that have been desensitized in the supplementary documents for the convenience of the researchers' review.

Authors,
Zhifei Xu , Zhigeng Pan ,Yan Wang5, Yichao Zhang, Pengfei Leng
